# Aerobic Exercise Training Prevents the Onset of Endothelial Dysfunction via Increased Nitric Oxide Bioavailability and Reduced Reactive Oxygen Species in an Experimental Model of Menopause

**DOI:** 10.1371/journal.pone.0125388

**Published:** 2015-04-29

**Authors:** Viviane A. V. N. Braga, Gisele K. Couto, Mariana C. Lazzarin, Luciana V. Rossoni, Alessandra Medeiros

**Affiliations:** 1 Department of Biosciences, Federal University of Sao Paulo, Santos, Sao Paulo, Brazil; 2 Department of Physiology and Biophysics, Institute of Biomedical Sciences, University of Sao Paulo, Sao Paulo, Sao Paulo, Brazil; University of Southampton, UNITED KINGDOM

## Abstract

**Objective:**

Previous studies have shown that estrogen deficiency, arising in postmenopause, promotes endothelial dysfunction. This study evaluated the effects of aerobic exercise training on endothelial dependent vasodilation of aorta in ovariectomized rats, specifically investigating the role of nitric oxide (NO) and reactive oxygen species (ROS).

**Methods:**

Female Wistar rats ovariectomized (OVX – n=20) or with intact ovary (SHAM – n=20) remained sedentary (OVX and SHAM) or performed aerobic exercise training on a treadmill 5 times a week for a period of 8 weeks (OVX-TRA and SHAM-TRA). In the thoracic aorta the endothelium-dependent and –independent vasodilation was assessed by acetylcholine (ACh) and sodium nitroprusside (SNP), respectively. Certain aortic rings were incubated with L-NAME to assess the NO modulation on the ACh-induced vasodilation. The fluorescence to dihydroethidium in aortic slices and plasma nitrite/nitrate concentrations were measured to evaluate ROS and NO bioavailability, respectively.

**Results:**

ACh-induced vasodilation was reduced in OVX rats as compared SHAM (Rmax: SHAM: 86±3.3 vs. OVX: 57±3.0%, p<0.01). Training prevented this response in OVX-TRA (Rmax: OVX-TRA: 88±2.0%, p<0.01), while did not change it in SHAM-TRA (Rmax: SHAM-TRA: 80±2.2%, p<0.01). The L-NAME incubation abolished the differences in ACh-induced relaxation among groups. SNP-induced vasodilation was not different among groups. OVX reduced nitrite/nitrate plasma concentrations and increased ROS in aortic slices, training as effective to restore these parameters to the SHAM levels.

**Conclusions:**

Exercise training, even in estrogen deficiency conditions, is able to improve endothelial dependent vasodilation in rat aorta via enhanced NO bioavailability and reduced ROS levels.

## Background

The incidence of cardiovascular diseases such as hypertension is lower in young men and women compared to postmenopausal women [1, 2]. This is due to the fact that estrogen deficiency observed in postmenopausal women is linked to the onset of several changes in the cardiovascular system, such as lower baroreflex sensitivity [3], increased blood pressure [4] and arterial stiffening [5]. This increase in the incidence of cardiovascular disease in postmenopausal women is worrying, since cardiovascular diseases are a major cause of mortality [6].

Several studies show that estrogen exerts direct action on the vascular endothelium. Therefore, reduction of estrogen levels contribute to the deterioration in endothelial function [7, 8]. Indeed endothelial dysfunction, characterized by reduced endothelium-dependent vasodilation and/ or increased endothelium-dependent contraction, has been observed in both ovariectomized Wistar rats [9] and in spontaneously hypertensive rats (SHR) [8, 10]. This impairment of endothelial function has been associated with increased production of reactive oxygen species (ROS), prostanoid factors and a reduced nitric oxide (NO) bioavailability due to lower production and higher inactivation; these being the main triggers of endothelial dysfunction in an experimental model of the menopause [9, 10].

The increased production of ROS in ovariectomized rats has been attributed to increased activity of the enzyme NAD(P)H oxidase [9] and by the increased expression of cyclo-oxygenase-2 (COX-2) [11, 12]. According to some studies, estrogen deficiency contributes to increase expression of AT1 receptors and a greater activation of the angiotensin converting enzyme (ACE), both changes contributing to increase activation of angiotensin II (AngII) [13, 14]. Ang II is responsible for activating the NAD(P)H oxidase which in turn promotes increased production of ROS.

Aerobic exercise training has been considered an important non-pharmacological tool for the improvement of endothelial function. It is known that regular aerobic exercise promotes benefits that contribute to maintaining vasomotor function. Studies show that aerobic exercise training, in some models, is able to reduce the expression of the subunits of NAD(P)H oxidase, as well as increase the activity of antioxidant enzymes and contribute significantly to the increase in production NO [3, 15, 16]. However, it remains unknown if aerobic exercise is capable of promoting its benefits even in a chronic deficiency of estrogen. Thus, the present study hypothesized that aerobic exercise training would act as a non-pharmacological tool to reverse the impairments in endothelial function observed in estrogen deficiency.

## Methods

### Animal care

The experiments were performed with Female Wistar rats 8 weeks of age, obtained from Cedeme, Federal University of São Paulo, Brazil. Rats were housed under controlled environmental conditions (temperature, 22°C; 12-h dark period) and had free access to standard laboratory chow (Nuvital Nutrients, Brazil) and water. The rats were randomly assigned into 4 groups: sedentary ovariectomized (OVX), trained ovariectomized (OVX-TRA), sedentary control (SHAM) and trained control (SHAM-TRA). This study was carried out in accordance with National Research Council's Guidelines for the Care and Use of Laboratory Animals [17] and was approved by the Ethics and Research Committee (CEP) of the UNIFESP (CEP #0116/12).

### Ovariotomy

At 8 weeks of age rats were anesthetized (50mg/kg ketamine and xylazine 12mg/kg, intraperitoneal) and a small incision in the abdominal region was made. The ovaries were located and ligation of the oviducts was performed, including blood vessels. The oviducts were sectioned and ovaries removed. The muscles and skin were sutured and a dose of antibiotics was administered (Benzetacil 40 000 U/kg, IM).

### Exercise training

Aerobic exercise training was performed on a treadmill at a low-moderate intensity (50–60% of the initial maximum speed), 1 hour per day, 5 days per week for 8 weeks. All rats were familiarized to the procedures (10 minutes, using different speeds) for 1 week. The adjustment period began 1 week after ovariectomy. Exercise capacity, estimated by total distance run, was evaluated with an incremental test to exhaustion using a protocol with an initial velocity of 5m/min being intensified every 5 minutes with a speed of 5 m/min, until the moment when the animal was unable to maintain the speed [18]. Both sedentary and trained rats were subjected to a maximum speed test, and this test was performed at the beginning, middle, and end of the experiment in order to assess the intensity of exercise and physical capacity.

### Indirect blood pressure recording

Blood pressure and heart rate were performed by tail plethysmography, using a specific system for rats (Visitech Systems: BP-2000—Series II—Blood Pressure Analysis System). Rats were acclimatized to the apparatus during daily sessions over 4 days, one week before starting the experimental period. The measurement was performed once a week throughout the experimental period, on days that trained groups were not subjected to exercise training. The average values for systolic blood pressure were subsequently obtained from ten sequential cuff inflation-deflation cycles.

### Tissue and plasma samples

24 hours after the last bout of aerobic exercise training, the rats were sacrificed by decapitation. Blood, thoracic aorta, soleus, plantaris and gastrocnemius muscles, as well as retroperitoneal fat, were removed immediately. In order to minimize the possible influences of estrogen levels in the SHAM rats, the sacrifice of SHAM rats occurs only in the metestrus phase of the cycle, since this phase is not ovulatory phase [19].

### Vascular Reactivity Study

Thoracic aortic segments (4 mm) were stripped of adherent tissue and mounted between two parallel wire hooks in an isolated tissue chamber containing Krebs-Henseleit solution (in mM: NaCl 115; KCl 4.7, MgSO_4_ 1.2; KH_2_PO_4_ 1.5; NaHCO_3_25; CaCl_2_ 2.5; glucose 11.1), gassed with 95% O_2_ and 5% CO_2_, and maintained resting tension of 2 g at 37°C at pH 7.4 as described previously [20, 21]. Isometric tension was recorded using an isometric force transducer (TIM 0:25, Make: Projects AVS) connected to an acquisition system (AQAD, Brazil).

After 60 minutes equilibration, the aortic rings were exposed to a pre-contraction with norepinephrine (NE, 10^-6^ M), and then concentration—response curves were made to the acetylcholine (ACh, 10^-9^ a 10^-4^ M), endothelium-dependent vasodilator, and to the sodium nitroprusside (SNP, 10^-11^ a 10^-5^ M), nitric oxide donor.

The role of NO in the ACh-induced response was evaluated by incubating some aortic rings with the non-selective NOS inhibitor L-nitro-L-arginine methyl ester (L-NAME 10^-4^M), added 30 minutes before the construction of the concentration—response curves to ACh. For concentration—response curve to ACh, the agonist concentration log resulting in 50% of the Rmax (log EC50) were calculated using non-linear regression analysis (GraphPad Prism software, USA). To compare the effect of NO in the ACh-induced response some results were expressed as ‘differences of area under the concentration—response curves' (dAUC) in control and experimental situations. AUCs were calculated from the individual concentration—response curves and the differences were expressed as a percentage of the AUC of the corresponding control.

### Measurement of plasma nitrite/nitrate

Plasma nitrite/nitrate levels were measured by the Griess method. The samples were centrifuged in tubes of ultra-filtration (Millipore) to separate hemoglobin. The preparation of the samples, standards, and cofactors of Griess reagent were carried out according to the description of commercially available kits (Nitrate/Nitrite Colorimetric Assay kit, Cayman Chemical, Ann Arbor, MI, USA). Assays were performed in duplicate and read in a spectrophotometer plate at wavelength 540–550nm [22].

### Generation ROS

The oxidative fluorescent dye dihydroethidium (DHE) was used to evaluate the *in situ* production of ROS [23]. Transverse aortic sections (10 μm) obtained in a cryostat were incubated at 37°C for 10 min with phosphate buffer containing DTPA (100 mM). Afterwards, each tissue section was incubated with DHE (2 μM) in a light-protected humidified chamber at 37°C for 30 min. Some aortic slices were incubated with a mimic of superoxide dismutase (SOD), the MnTMPyP (25 μM) for 30 minutes at 37°C. Negative control sections received the same volume of buffer without hydroethidine. Images were obtained using an optical microscope (Eclipse 80i, Nikon, Japan) equipped with filter to rhodamine and camera (DS—U3, Nikon, Japan), using a 20x objective. The images were analyzed with the Image J software (National Institute of Health, Bethesda-MD, USA) by the integrative density of the fluorescence observed in the artery. The results were expressed as the delta of basal integrative density minus in the presence of MnTMPyP.

### Statistical Analysis

The data are expressed as mean ± standard error of the mean. The effect of aerobic exercise training was tested by analysis of variance (ANOVA) one or two-way, as appropriate. If statistically significant differences were detected by ANOVA, post-hoc Duncan test was performed. The normality test was performed and all data followed a normal distribution. The significance threshold was set to p≤0.05.

## Results

### Biometric parameters and hemodynamic

Biometric parameters and hemodynamic data are presented in Table 1. The OVX rats increased body mass and retroperitoneal fat as compared to SHAM rats. However, the OVX rats showed a decrease in soleus and plantaris muscle mass. On the other hand, aerobic exercise training caused a reduction of body mass and retroperitoneal fat and maintain the soleus muscle mass and increase the plantaris muscle mass as compared to SHAM training rats. Moreover, aerobic exercise training did not change these parameters in SHAM animals (Table 1).

**Table 1:** Anthropometric and Hemodynamic data from SHAM, SHAM-TRA, OVX and OVX-TRA rats.

	SHAM	SHAM-TRA	OVX	OVX-TRA
Weight (g)	267±6.4	258±5.5	307±7.9*	287±6.0* ^#^
Soleus (mg/g)	0.060±0.002	0.061±0.002	0.050±0.002*	0.061±0.002^#^
Plantaris (mg/g)	0.086±0,003	0.089±0.003	0.084±0.002	0.101±0.01^$^ ^#^
Retroperitoneal fat (mg/g)	37.90±3.69	31.92±1.79	48.10±2.78*	34.07±2.82^#^
SBP (mmHg)	143±3.2	147±2.0	169±4.2*	145±3.3^#^

* p<0.05 OVX and OVX-TRA vs. SHAM and SHAM-TRA;

^#^ p<0.05 OVX-TRA vs. OVX,

^$^ p<0.05 OVX-TRA vs. SHAM.

Ovariectomy increased systolic blood pressure in the OVX rats compared to SHAM rats. Exercise training prevented the increase in systolic blood pressure in OVX-TRA rats, not altering the blood pressure values in SHAM-TRA rats.

### Vascular Reactivity

**Fig 1:**
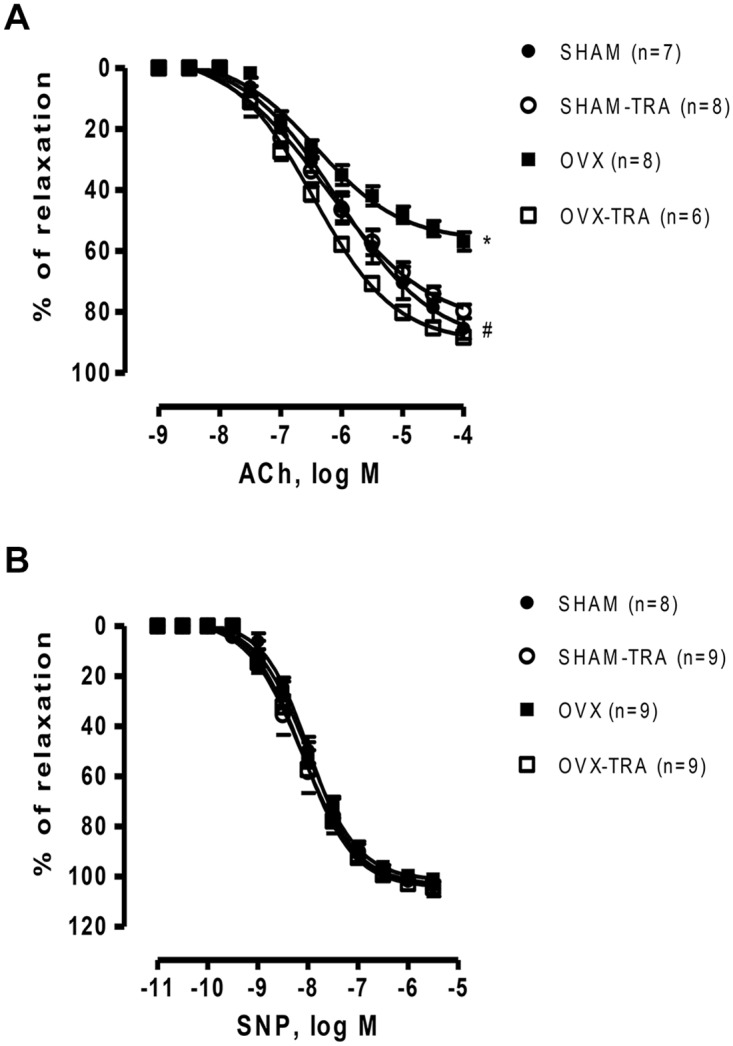
Concentration—response curve to acetylcholine (A) and sodium nitroprusside (B) in aortic rings from SHAM, SHAM-TRA, OVX and OVX-TRA. The numbers shown in the legend represent the number of animals analyzed in each group. Results are expressed as means±SEM. Two-way ANOVA: *p<0.01 in comparison to SHAM, # p<0.01 in comparison to OVX.

The NE-induced (10^-6^ M) pre-contraction tonus was similar in all groups. The endothelium-dependent vasodilation induced by ACh was reduced in OVX aortas as compared to SHAM (Fig 1A) (Rmax: SHAM: 86±3.3 vs. OVX: 57±3.0%, p<0.01). Aerobic exercise training improved vasodilation induced by ACh in aorta from OVX-TRA rats as compared to SHAM-TRA rats (Fig 1A) (Rmax: SHAM-TRA: 80±2.2% vs. OVX-TRA: 88±2.0%, p<0.01), while it did not change the ACh-induced relaxation in aorta from SHAM-TRA as compared to SHAM (Fig 1A). However, no significant change in the vasodilator response induced by SNP was observed among groups (Fig 1B).

**Fig 2:**
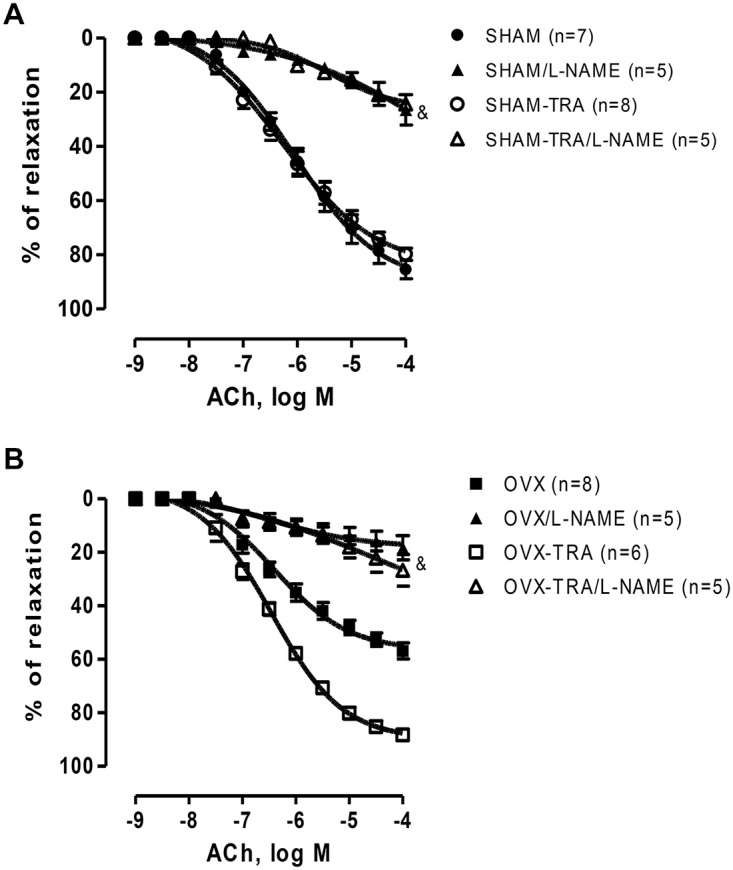
Effect of L-NAME (100 μM) on the concentration—response curve of ACh in aortic rings from SHAM vs SHAM-TRA (A) and OVX vs OVX-TRA (B). The numbers shown in the graphs represent the number of animals analyzed in each group. Results are expressed as means±SEM. Two-way ANOVA: & p<0.01 in comparison to control; *p<0.01 in comparison to SHAM, # p<0.01 in comparison to OVX.

The L-NAME incubation reduced the ACh-induced relaxation in all groups (Fig 2A and 2B). The area under the curve (dAUC %) between the ACh-induced relaxation in the presence and absence of L-NAME among groups suggested that OVX aortas present a reduced NO response as compared to SHAM aortas; however, aerobic training was effective in improve NO participation in the ACh vasodilation (Table 2). Taken together, the vascular reactivity results suggested that exercise training increased NO bioavailability in OVX aortas.

**Table 2:** Effect of L-NAME (100 μM) on the concentration—response curve of ACh in aortic rings from SHAM, SHAM-TRA, OVX and OVX-TRA: area under the curve (dAUC %).

	SHAM	SHAM-TRA	OVX	OVX-TRA
dAUC %	42.27±1.52	43.23±0.56	24.28±2.53*	52.07±1.28* ^#^

* p<0.05 OVX and OVX-TRA vs. SHAM and SHAM-TRA;

^#^ p<0.05 OVX-TRA vs. OVX. dAUC are expressed as a percentage of the corresponding AUC for L-NAME aortic rings.

### Nitrite/nitrate plasma levels

**Fig 3:**
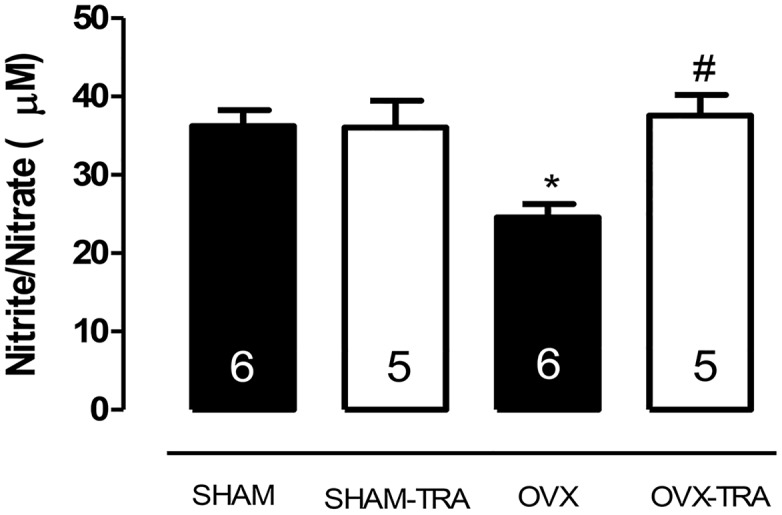
Nitrite/nitrate plasma levels from SHAM, SHAM-TRA, OVX and OVX-TRA rats. Results are expressed as means±SEM. The numbers shown in bars represent the number of animals analyzed in each group. Two-way ANOVA: *p<0.01 in comparison to SHAM, # p<0.01 in comparison to OVX.

In line with the vascular reactivity results, the nitrite/nitrate plasma levels were reduced in OVX rats as compared to SHAM animals; exercise training increased the nitrite/nitrate levels in OVX-TRA rats, but did not change it in SHAM-TRA rats (Fig 3).

### Generation ROS

**Fig 4:**
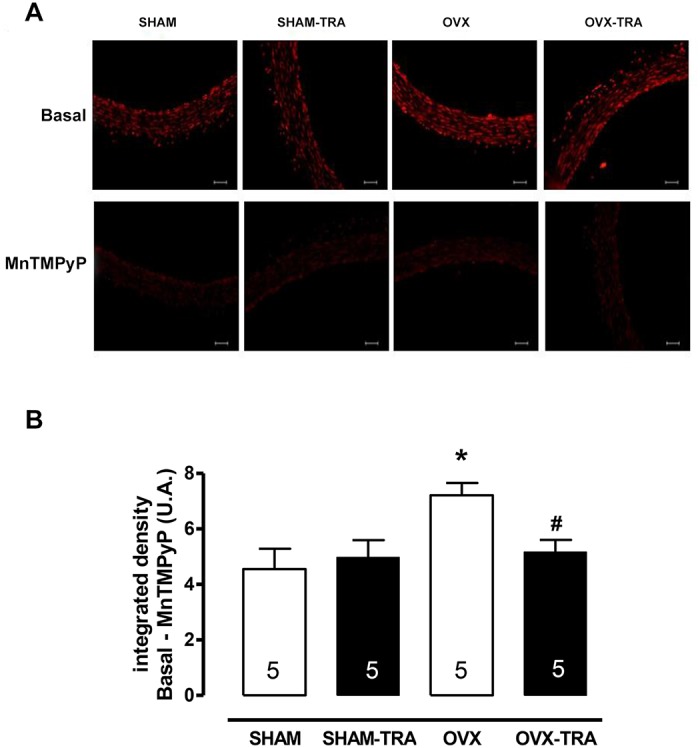
Representative images of aortic slices from SHAM, SHAM-TRA, OVX and OVX-TRA rats, in the absence (upper panel) and presence (lower panel) of the superoxide dismutase mimetic, MnTMPyP (A). Quantification of fluorescence intensity emitted by DHE oxidation in aortic slices in the absence and presence of the MnTMPyP as an indicative of reactive oxygen species in SHAM, SHAM-TRA, OVX and OVX-TRA rats (B). The numbers shown in bars represent the number of animals analyzed in each group. Results are expressed as means±SEM. Two-way ANOVA: *p<0.01 in comparison to SHAM, # p<0.01 in comparison to OVX.

To assess the production of ROS in the aorta, we used fluorescence emitted by DHE in the presence and absence of the mimic of superoxide dismutase (SOD), the MnTMPyP (25 μM), as an indicative of superoxide anion bioavailability. As seen in Fig 4, the intensity of fluorescence emitted by DHE was higher in the aorta of OVX rats when compared to SHAM rats, indicating an increased level of ROS. Aerobic exercise training reduced ROS levels in the aorta of OVX-TRA to SHAM levels and did not alter it in SHAM-TRA aortas. The mimic of superoxide dismutase (SOD), the MnTMPyP, blocked the differences among aortas of studied groups, suggesting that the superoxide anion is the major ROS produced in OVX aorta, which is modulated by exercise training (Fig 4).

## Discussion

The present results suggested that 8 weeks of treadmill aerobic training is a health condition to restore biometric parameters, blood pressure and endothelial-dependent vasodilation in ovariectomized rats. The improved endothelial-dependent vasodilation in aorta from ovariectomized rats is associated to increased NO and reduced superoxide anion bioavailability.

The menopause experimental model has been widely used in the literature, since studies show that in young adult female rats (6 to 12 weeks) the removal of the ovaries leads to restriction of ovarian hormones similar to that observed in postmenopausal women [3, 24]. The relationship between reduced levels of estrogen and the appearance of changes in the cardiovascular system has been often widely described in the literature. Accordingly, estrogen deficiency has been shown to contribute to the development of hemodynamic and vascular changes, such as an increased blood pressure and endothelial dysfunction.

Besides the changes in the cardiovascular system, it is noteworthy that estrogen deficiency also contributes to the onset of changes in body composition in both humans and animals [24–26]. This study shows that estrogen deficiency was associated with an increase of greater magnitude in body mass, reduced muscle mass, and especially the accumulation of adipose tissue in sedentary ovariectomized rats. On the other hand these changes were minimized by exercise training. Several studies have showed that exercise training contributes to the reduction of body mass and visceral adipose tissue [27–29].

In the present study, it was observed that lack of estrogen was responsible for the increased systolic blood pressure in ovariectomized rats, which corroborates the literature, since the increased blood pressure after menopause has been observed in several studies [4, 10]. Nevertheless, aerobic exercise training significantly reduced the blood pressure in the ovariectomized rats towards control group levels. Hypotension post-aerobic exercise training in hypertension has been a consistent finding in the literature [22, 30–33]. In fact, aerobic exercise training promotes acute and chronic adaptations in the cardiovascular system [34–36].

Besides the increased blood pressure, estrogen deficiency also triggers endothelial dysfunction in aorta of ovariectomized rats, and this dysfunction was characterized by reduced endothelium-dependent vasodilation. At the present study we decided to use the aortic rings as a model to study vascular function as the main endothelium dependent vasodilator factor is NO [37]. The results of this study confirm that endothelial dysfunction arising from estrogen deficiency (an experimental model of menopausal) occurs through a lower NO bioavailability and increased levels of ROS. Our results corroborate findings in the literature, since estrogen deficiency contributes to the onset of endothelial dysfunction both in experimental models [9] and in humans [38]. Yung et al (2011) [9] observed that ovariectomized rats showed a progressive deterioration of endothelium-dependent vasodilation induced by ACh, and this vasodilation was impaired from the 4th week after ovariectomy. The same can be observed by Widder et al (2003) [39], since it was possible to observe a significant reduction in endothelium-dependent vasodilation induced by ACh in the aorta of ovariectomized rats.

Supporting the hypothesis of the present study, 8 weeks of aerobic exercise training was effective in reducing blood pressure and preventing the onset of endothelial dysfunction in ovariectomized rats. In fact, the training restored the endothelium-dependent vasodilation induced by ACh in ovarectomized rats, result that is in line with the literature. Figard et al. (2006) [40] showed that isometric strength exercise training contributes to increased endothelium-dependent vasodilation in ovariectomized rats and that this increase in vasodilation is due to greater activation of NOS, cyclooxygenase products, calcium pathways and endothelium-derived hyperpolarizing factors. In addition, Claudio et al (2013) [41] demonstrated that swimming training improved coronary vasodilation by enhanced NO bioavailability in ovariectomized rats. On the other hand, Pierce et al. (2011) [42] showed that 8 weeks of daily brisk walking was not able to improve the brachial artery flow-mediated dilation in postmenopausal women. Taken together these results suggest that to improve vasodilation in estrogen deficiency is necessary prolonged and more intense exercise training.

Evidence shows that endothelial dysfunction arising from the reduction in estrogen levels occurs through the unbalance between NO and ROS bioavailability [8]. Indeed, when we analyzed the modulatory activity of NO by incubation of aortic rings with L-NAME, a non-selective inhibitor of NOS, we found that ACh-induced vasodilation was reduced in all groups. Thus OVX group showed a lower NO modulation when compared to SHAM group and training was able to restore the NO response in ovarectomized rats to control levels. In agreement with our functional data, we can see that the nitrate/nitrite plasma levels were reduced in OVX rats, and in an interesting manner aerobic training caused an increase in these levels in OVX-TRA rats. These results are supported by previously studies demonstrating that isometric strength exercise training [30] and swimming training [41] increased NO bioavailability in aorta and coronary arteries from ovarectomized rats. Based on the above, we can assign the prevention of endothelial dysfunction group of OVX-TRA to increase the NO bioavailability, and consequently the higher modulatory activity of NO.

Aerobic exercise training is responsible for increasing the expression and activity of eNOS [41, 43]. The activation of eNOS after aerobic exercise training occurs by shear stress and through the activation of PI3K/Akt/PKB and PKA pathways [44]. However, it is well known that the increased blood flow to the skeletal muscle during physical exercise also alters the release and synthesis of endothelial-dependent prostaglandins, which aids in increasing the vasodilation induced by acetylcholine [45]. In fact, Koller et al (1995) [46] showed that the practice of daily exercise promotes increased flow-dependent dilation of skeletal muscle arterioles of rats and that this increase in arteriolar dilation results from a greater release of both nitric oxide and prostaglandins.

Reinforcing our results, we can see that the OVX group showed an increase in superoxide level in the vessel. As we have seen the development of endothelial dysfunction caused by estrogen deficiency is also associated with an increased level of ROS. In the artery, ROS are produced by several enzymatic systems including: NADPH oxidase, COX-2, xanthine oxidase, uncoupled eNOS and mitochondrial electron transport chain [47, 48]. There are several pathways that may be contributing to the increased oxidative stress in ovariectomized rats. According to Wassmann et al (2001) [8], increased level of ROS in the vessel of ovariectomized rats occurs via increased activation of the Ang II, since estrogen deficiency provides a significant increase in the expression of AT1 receptors and increased vascular response in the presence of Ang II. The increased activity of Ang II in the vessel contributes to increased activation of the enzyme NADPH oxidase, which is one of the major source of ROS in the vessel wall. Camporez et al (2011) [49] showed that estrogen deficiency also promotes an increased expression of gp91phox and 22phox subunits of NADPH oxidase. Another pathway that may be contributing to the increased oxidative stress in ovariectomized rats is the pathway of cyclo-oxygenase, since some studies showed that ovariectomy is responsible for the increased COX-2 expression and increased synthesis of thromboxane [11, 12]. Dong et al (2013) [50] showed that the inhibition of COX-2 as well the antagonism of the TP receptor restore the impaired ACh-induced vasodilatation in renal artery of ovariectomized rats, suggesting an involvement of COX-2 and TP receptor on ROS generation and endothelial dysfunction triggered by estrogen deficiency.

Interesting, the present results demonstrated that aerobic exercise training reduced the bioavailability of the superoxide anion in aortas from ovariectomized rats. Adams et al (2005) [15] observed that aerobic exercise in patients with coronary artery disease reduces both the mRNA and the protein expression of NADPH oxidase. The redox status is the balance between the generation of ROS, as described above, and its degradation by antioxidant enzyme systems, including superoxide dismutases, catalase, glutathione peroxidase [47, 48]. The aorta of ovariectomized rats present a reduction in the expression of Cu/Zn-SOD but not in the expression of Mn-SOD [35], while neither SOD nor catalase changed in coronary artery of ovariectomized rats [41]. Data in the literature also show that aerobic exercise training can provide an increased expression of antioxidant enzymes in ovariectomized rats. The study of Claudio et al (2013) [41] observed that after 8 weeks of swimming training promoted an increase in SOD-1 expression and catalase in coronary artery ovariectomized rats. Similar results can be observed in the study of Irigoyen, et al. (2005) [3] since after a period of exercise training, it was possible to observe an increase in myocardial SOD and also in skeletal muscle catalase of ovariectomized rats. It is believed that the largest modulation of the expression of SOD after exercise training would be mediated endothelial mechanical stress, since the shear stress exerted by physical exercise would be able to enhance expression of the Cu/Zn SOD in endothelial cells from human aorta [51].

However, on limitation of the present study was the impossibility to identify what is or are the pathway(s) involved in the enhanced bioavailability of superoxide anion present in the aorta of the ovariectomized rats and the pathway by which exercise modulates this response.

## Conclusion

In summary, this study confirms a reduced NO bioavailability and increased production of ROS is associated with the onset of endothelial dysfunction in an animal model of estrogen deficiency. Furthermore our results demonstrate the important role of aerobic exercise training as a non-pharmacological treatment for improving endothelial function, lowering blood pressure, and decreasing the production of ROS in the aorta of ovariectomized rats.
